# Supplementary material for: Risk assessment and perspectives of local transmission of chikungunya and dengue in Italy, a European forerunner
Source: Nat Commun. 2025 Jul 7;16:6237. doi: 10.1038/s41467-025-61109-1 (PMC12234714; doi:10.1038/s41467-025-61109-1)
Supplement: Supplementary file 1 — Supplementary Information [file 41467_2025_61109_MOESM1_ESM.pdf]

SUPPLEMENTARY INFORMATION

Risk assessment and perspectives of local transmission of chikungunya and dengue in Italy, a European forerunner

Surveillance and control of CHIKV and DENV infections in Italy **2**

Statistical analysis of imported cases **5**

Temporal trends in case importation **5**

Temporal trends in notification delays **7**

Difference in notification delays between autochthonous and imported cases **9**

Transmissibility from surveillance of human cases ***11***

The risk of onward transmission and the likelihood of experiencing local outbreaks after case importation ***12***

References ***18***

Surveillance and control of CHIKV and DENV infections in Italy

Surveillance of DENV and CHIKV in Italy is defined in an integrated national surveillance and response plan[1] that adopts the current EU case definition of these diseases[2]. Data on the presence and the abundance of *Aedes albopictus*, the main competent vector present in Italy for these diseases, including data on seasonal fluctuations of the beginning and end of its trophic activity is collected by monitoring sentinel sites (Hot Spots) using ovitraps and/or BG Sentinel® traps, the number and location of which is established with the advice of expert entomologists and other competent local health authorities. This system is also designed to detect the possible introduction of new invasive Aedes mosquitoes, such as *Aedes aegypti*.

Actions to reduce the presence of man-made *Aedes* breeding sites and limit abundance, in the absence of evidence of CHIKV/DENV human infection, is performed routinely mainly to reduce nuisance through communication campaigns and local mosquito control programs.

Surveillance of human cases of DENV and CHIKV is mandatory and active throughout the year. It is enhanced during the season of vector activity when any suspected human case (imported or locally acquired) needs to be notified by medical doctors to the local health authorities (LHA) within 12 hours of detection. This triggers immediate investigation of the case (epidemiological and entomological) and, from June to October the activation the vector control interventions within 24 hours of notification, to be carried out extensively, both on public and private land, and in parallel with larvicides and environmental remediation treatment. Measures focus on the place of residence of the case and, in case of suspected local transmission, in an area of approximately 200 meters radius around the place where the human case presumably was exposed to the infection. Entomological investigations can include:

- positioning of a BG Sentinel® trap with an attractant (Lure, Octenol, etc.) or activated with CO2 inside or near the home of the human case, operating from early morning to dusk;
- evaluation of the presence of adults by direct observation;
- verification of the presence of active larval outbreaks in both public and private areas.

In case local transmission is suspected, xenomonitoring in pools of local adult mosquitoes captured in proximity of the case/s is performed in order to confirm vector-borne transmission.

Vector control measures are based on three main activities:

- Vector control of the affected area with insecticides, giving priority to adulticide interventions, both on public land and on private property. This can include spatial knockdown interventions and residual interventions;
- Research and elimination of peri-domestic larval breeding sites, with "door-to-door" inspection of homes included in the area. Larvicidal treatments are reserved for non-removable breeding sites. In urban environments, e.g. for the treatment of manholes, it is possible to choose between biological larvicides based on spore-forming bacteria alone, or in association, and growth regulators (IGR) or similar products. Alternatively, it is possible to use physical means such as monomolecular films.
- Strengthening of information campaigns targeting residents.

Clinical samples are sent to the Regional and/or National Reference Laboratories for Arboviruses for laboratory confirmation as previously described[3–6] by both molecular and

serological assays according to a predefined diagnostic algorithm[1]. Most cases are usually confirmed through direct virus detection methods, also due to high percentages of cross reactivity among flaviviruses. Control measures on Substances of Human Origin (SoHO), are triggered when local transmission is confirmed.

Table S1 summarizes the demographic and clinical characteristics of DENV and CHIKV laboratory-confirmed infections notified to the Italian National Surveillance system between 2006 and 2023. Fig. S1 shows the country of likely exposure of travel-related DENV and CHIKV cases as identified during epidemiological interviews.

Table S1. Demographic and clinical characteristics of DENV and CHIKV laboratory-confirmed infections notified to the Italian National Surveillance system (n=2,058), Italy, 2006 and 2023.

	**CHIKV (n=530)**		**DENV (n=1,528)**	
	**n**	**%**	**n**	**%**
**Age group (year)**				
<20	42	8.0	140	9.2
20-39	111	21.2	737	48.5
40-59	165	31.5	485	31.9
60+	206	39.3	157	10.3
**Sex**				
Female	256	48.3	679	44.5
Male	274	51.7	848	55.5
**Symptom**				
Fever	499	94.1	1424	93.2
Asthenia	362	68.3	1111	72.7
Arthralgias	484	91.3	956	62.6
Skin rash	310	58.5	708	46.3
Diarrhea	33	6.2	65	4.2
Vomit	32	6.0	39	2.6
Itching	38	7.2	15	1.0
Nausea	17	3.2	24	1.6
Conjunctivitis	15	2.8	24	1.6
Thrombocytopenia	1	0,2	31	2.0
Myalgia	24	4.5	1	0.1
Haemorrhagic symptoms	2	0.4	23	1.5
Headache	22	4.2	0	0.0
Meningoencephalitis	5	0.9	10	0.6
Gastric/Abdominal pain	0	0.0	13	0.9
Photophobia	7	1.3	2	0.1
Lymphadenopathy	3	0.6	6	0.4
Edema	7	1.3	2	0.1
Other neurological symptoms	3	0.6	3	0.2
Hepatosplenomegaly	1	0.2	3	0.2

Syncope	0	0.0	4	0.3
Arthritis	2	0.4	0	0.0
Other symptoms	4	0.7	33	2.2

Fig. S1. Countries of exposure reported by imported DENV and CHIKV cases. Administrative boundaries of the countries of the world © Esri; Garmin International, Inc.; U.S. Central Intelligence Agency; International Organization for Standardization (ISO).

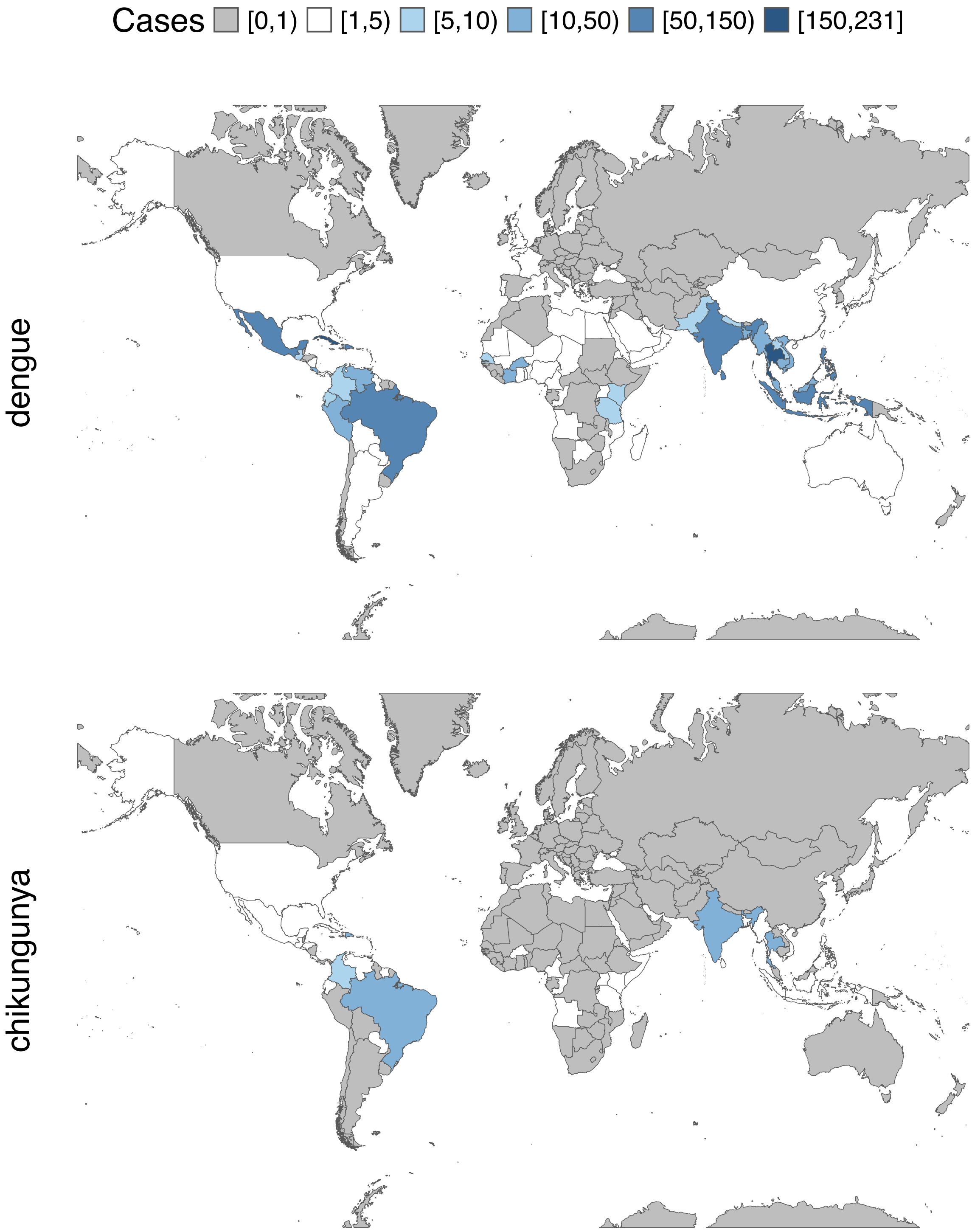

Statistical analysis of imported cases

We analyzed the temporal trends of imported cases and reporting delays for both chikungunya and dengue by means of Generalized Additive Model (GAM) incorporating an autoregressive term and Generalized Additive Mixed Model (GAMM), respectively, in a frequentist framework using the function *gamm* implemented in the R package *mgcv* (R Project for Statistical Computing, software version 4.3.2)[7–9]. We considered additive models over linear due to their flexibility as we hypothesized that the response variable (i.e., the number of imported cases or reporting delays) would depend linearly on one or more smooth functions of covariates rather than the covariates themselves (e.g. the number of imported cases would display a seasonal trend rather than a monotonic relationship with months).

Temporal trends in case importation

For each disease (*d* = dengue or chikungunya), we computed the number of imported cases per month ($I_{d,m}$) from July 2006 to November 2023 (*m* = 1…209), aggregated by date of symptom onset. We considered $I_{d,m}$ as the dependent variable and we assumed it to follow a Poisson distribution with log link. In the model, we incorporated two qualitative independent variables: the specific disease (*D*, modeled as a binary variable: 0 for dengue and 1 for chikungunya) affecting the imported cases and whether importation occurred during travel restrictions (*C*, modeled as a binary variable assuming value 0 when not occurring during travel restrictions and 1 otherwise) put in place during the COVID-19 crisis (i.e., symptom onset between February 2020 and December 2020[10]). Additionally, we considered two quantitative independent variables: the year (*Year*) and month (*Month*) of symptom onset, modeled as penalized cubic splines (*f*) and penalized cyclic cubic splines (*g*), respectively. The interaction between these terms and the disease was also considered for a total of four smoothing functions (month & dengue, month & chikungunya, year & dengue, year & chikungunya), thus allowing each disease to have a different relationship between cases and month of importation, as well as allowing each disease to have a different relationship between cases and year of importation. Finally, an autoregressive term of order two (ϕ_1, ϕ_2) was included to deal with the temporal autocorrelation detected in the model residuals.

The model equations are the following:

$$I_{d,m} \sim Poisson(\lambda_{d,m})$$
$$E(I_{d,m}) = \lambda_{d,m}\ ;\ Var(I_{d,m}) = \lambda_{d,m}$$
$$log(\lambda_{d,m}) = \beta_0 + \beta_1 D_{d,m} + \beta_2 C_m + f_d(Year_m) + g_d(Month_m) + \eta_{d,m}$$
$$\eta_{d,m} = \phi_1 \eta_{d,m-1} + \phi_2 \eta_{d,m-2} + \varepsilon_{d,m}$$
$$\varepsilon_{d,m} \sim Norm(0, \sigma^2)$$

where β_0, β_1 and β_2 are the regression parameters to be estimated along with f_d, g_d, ϕ_1, and ϕ_2.

In the analyzed dataset, the number of imported cases of chikungunya was found to be lower compared to dengue, as shown by the negative mean estimate and 95% confidence interval of

the regression parameter β_1 (Table S2). As expected, model results show that the number of imported cases dropped during travel restrictions imposed to control the COVID-19 pandemic, as confirmed by the negative mean estimate and 95% confidence interval of the regression parameter β_2. We found evidence of a non-linear trend in the number of importations for both diseases as estimated by the splines modeling annual and seasonal trends. Model assumptions were verified by assessment of temporal dependency, concurvity measures (Table S3), and visual inspection of model residuals (Fig. S2)[11]. Concurvity arises when a smooth term of the model might be approximated by one or more of the other smooth terms within the same model and can be interpreted as a generalization of co-linearity.

Table S2. Estimated model parameters in the analysis of temporal trends of imported cases. The tests used to compute the reported p-values are the Wald-type tests implemented in the function *gamm* from the R package *mcgv*.

Regression parameters	***Estimate***	***95% Confidence Intervals***	***p-value***
Intercept (β_0)<br>Disease: dengue<br>COVID-19: No	1.349	1.202, 1.496	<0.0001
Disease: chikungunya (β_1)	-2.076	-2.411, -1.740	<0.0001
COVID-19: Yes (β_2)	-2.710	-3.586, -1.833	<0.0001
Splines	***Estimated degree of freedom***	***F***	***p-value***
f_{dengue} (Year)	8.239	44.947	<0.0001
$f_{chikungunya}$ (Year)	7.228	7.258	<0.0001
g_{dengue} (Month)	6.991	60.217	<0.0001
$g_{chikungunya}$ (Month)	3.709	2.043	0.00089
Correlation Structure: ARMA(2,0)	***Estimate***		
ϕ_1	0.223		
ϕ_2	0.032		
σ^2	1		

Table S3. Concurvity measures. Indices of concurvity as obtained from 'concurvity' function in mgcv R package. A value of 0 indicates no issues related to concurvity, while a value of 1 means a lack of identifiability. The three indices ("worst", "observed", and "estimate") represent the extent to which each smoothing term can be entirely described by one or more other terms in the model.

	Parameters	f_{dengue}(Year)	$f_{chikungunya}$(Year)	g_{dengue}(Month)	$g_{chikungunya}$(Month)
worst[a]	0.521	0.224	0.224	0.019	0.019
observed[b]	0.521	0.018	0.028	0.011	0.003

estimate [c]	0.521	0.029	0.029	0.003	0.003

All indices are based on the idea that each smoother *f* can be decomposed into a part (*g*) that lies entirely within the space of one or more terms in the model, and a remainder part that is completely within *f*'s own space. Concurvity issues arise if *g* constitutes a large part of *f*. Indices are all based on the square of ||*g*||/||*f*||, that is the ratio of the squared Euclidean norms of the vectors of *f* and *g* evaluated at the observed covariate values.

[a]It is the largest value that the square of ||*g*||/||*f*|| could take for any coefficient vector. This is the most pessimistic measure, as it looks at the worst case irrespective of data.

[b]It returns the value of the square of ||*g*||/||*f*|| according to the estimated coefficients.

[c]It is the squared F-norm of the basis for *g* divided by the F-norm of the basis for *f*.

Fig. S2. Model residuals observed in the analysis of the temporal trends of imported cases.

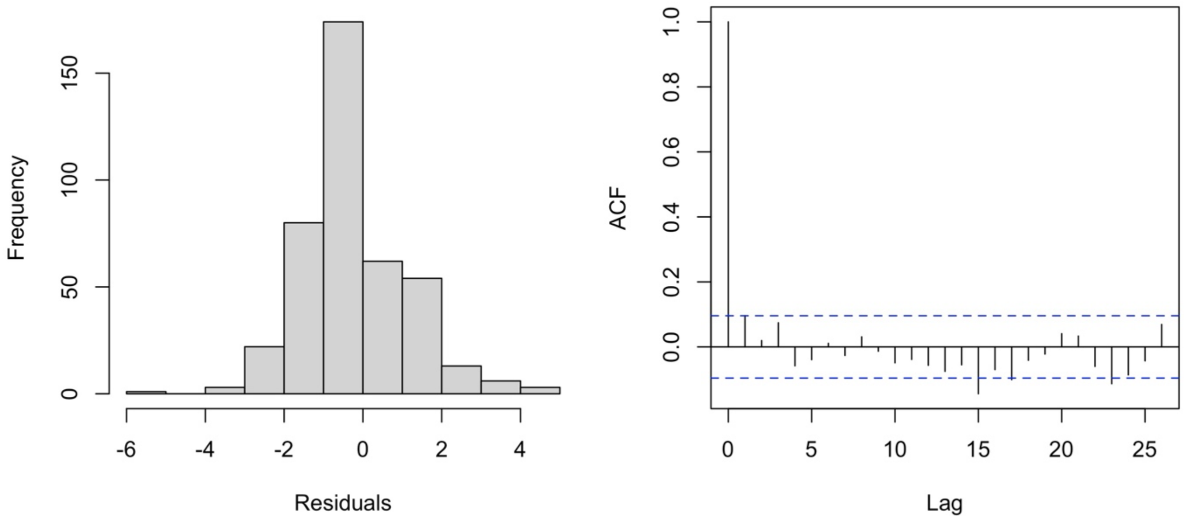

Temporal trends in notification delays

For each imported case *j* (*j*=1 … 1577), we defined the notification delay R_j as the time between their symptom onset and their reporting date to the Italian National Institute of Health. We considered R_j as the dependent variable and we assumed it to follow a Negative Binomial distribution with log link. We included in the model one qualitative independent variable: the specific disease (*D*, modeled as a binary variable: 0 for dengue and 1 for chikungunya) affecting the imported cases. Additionally, we included in the model two quantitative independent variables: the year (*Year*) and month (*Month*) of symptom onset, modeled as penalized cubic splines (*f*) and penalized cyclic cubic splines (*g*), respectively. The interaction between these terms and the disease was considered as for the previous GAM. Finally, we considered the region of importation as a random effect and an autoregressive term of order 1 (ϕ_1) to deal with the autocorrelation detected in the model residuals.

The model equations are the following:

$$R_j \sim NegBin(\mu_j, \theta)$$
$$E(R_j) = \mu_j \; ; \; Var(R_j) = \mu_j + \mu_j^2/\theta$$
$$log(\mu_j) = \beta_0 + \beta_1 D_j + f_d(Year_j) + g_d(Month_j) + \eta_j$$

$$\eta_j = \phi_1 \eta_{j-1} + \varepsilon_j$$
$$\varepsilon_j \sim Norm(0, \sigma_r^2)$$

where β_0 and β_1 are the regression parameters to be estimated along with f_d, g_d, ϕ_1, and θ. In the analyzed dataset, we found evidence against the hypothesis of a comparable notification delay between dengue and chikungunya, with the latter showing on average a 1.62 (95% confidence interval: 1.37–1.91) times longer notification delay (Table S4).

Table S4. Estimated model parameters in the analysis of notification delays. The tests used to compute the reported p-values are the Wald-type tests implemented in the function *gamm* from the R package *mcgv*.

Regression parameters	*Estimate*	*95% Confidence Intervals*	*p-value*
Intercept (β_0)<br>Disease: dengue	2.447	2.312, 2.582	<0.0001
Disease: chikungunya (β_1)	0.481	0.316, 0.645	<0.0001
Spines	***Estimated degree of freedom***	***F***	***p-value***
f_{dengue} (Year)	1	54.473	<0.0001
$f_{\text{chikungunya}}$ (Year)	1	1.244	0.26486
g_{dengue} (Month)	2.153	0.943	0.01401
$g_{\text{chikungunya}}$ (Month)	<0.001	<0.001	0.60956
Parameters	***Estimate***		
θ	0.710		
σ_r^2	0.843		
ϕ_1	0.062		

The model splines estimated a decreasing linear relationship between year and notification delay for dengue while no statistical support was found for a relationship between year and notification delays for chikungunya, as suggested by descriptive statistics on observed data (Table S5). Finally, evidence for a non-linear trend in the notification delay between months was found for dengue but not chikungunya (Table S5). Model assumptions were verified by assessment of temporal dependency, concurvity measures (Table S6), and visual inspection of model residuals (Fig. S3)[11].

Table S5. Observed notification delays (in days) in 2010, 2023, and during the periods 2006 – 2010 and 2017 – 2023.

Disease	*Year*	*N*	*Mean*	*Median*	*IQR*	*Range*
Dengue	2010	51	14	10	6 - 19.5	1 - 68
Dengue	2023	244	9.16	7	4 - 11	0 – 84
Chikungunya	2010	5	14.8	20	7 - 20	6 – 21
Chikungunya	2023	7	13.7	12	9 - 17	6 – 26
Dengue	2006 - 2010	74	13.8	9	6 - 19.8	1 - 68
Dengue	2017 - 2023	826	9.6	7	5- 11	0 - 88

Chikungunya	2006 - 2010	12	14.8	13	6.75 - 20	1 - 44
Chikungunya	2017 - 2023	50	17.1	10	6 - 14.8	2 - 88

Fig. S3. Model residuals observed in the analysis of notification delays.

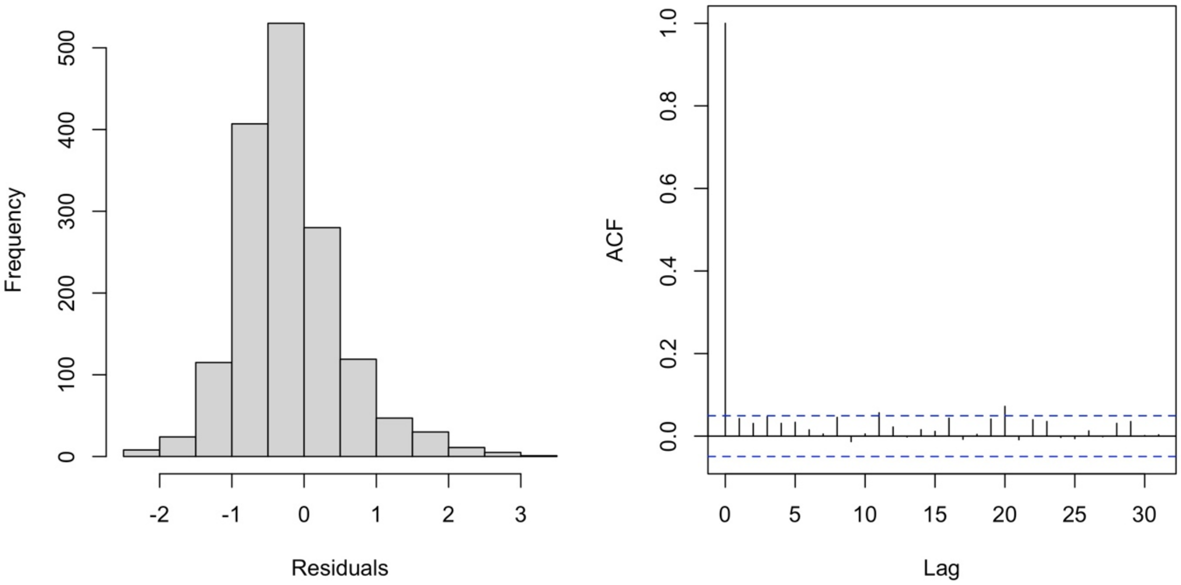

Table S6. Concurvity measures. Indices of concurvity as obtained from 'concurvity' function in mgcv R package. A value of 0 indicates no issues related to concurvity, while a value of 1 means a lack of identifiability. The three indices ("worst", "observed", and "estimate") represent the extent to which each smoothing term can be entirely described by one or more other terms in the model.

	Parameters	f_{dengue}(Year)	$f_{chikungunya}$(Year)	g_{dengue}(Month)	$g_{chikungunya}$(Month)
worst[a]	0.098	0.073	0.606	0.073	0.383
observed[b]	0.098	0.031	0.305	0.028	0.297
estimate[c]	0.098	0.030	0.257	0.022	0.169

All indices are based on the idea that each smoother *f* can be decomposed into a part (*g*) that lies entirely within the space of one or more terms in the model, and a remainder part that is completely within *f*'s own space. Concurvity issues arise if *g* constitutes a large part of *f*. Indices are all based on the square of ||*g*||/||*f*||, that is the ratio of the squared Euclidean norms of the vectors of *f* and *g* evaluated at the observed covariate values.

[a]It is the largest value that the square of ||*g*||/||*f*|| could take for any coefficient vector. This is the most pessimistic measure, as it looks at the worst case irrespective of data.

[b]It returns the value of the square of ||*g*||/||*f*|| according to the estimated coefficients.

[c]It is the squared F-norm of the basis for *g* divided by the F-norm of the basis for *f*.

Difference in notification delays between autochthonous and imported cases

To compare notification delays (R_S) between autochthonous and imported cases, we restricted our data to the subset of 608 cases notified in regions and years for which autochthonous transmission was documented (chikungunya: 2007 in Emilia-Romagna, 2017 in Lazio and

Calabria; dengue: 2020 in Veneto, 2023 in Lazio and Lombardy). We analyzed the notification delays of imported and autochthonous cases for both chikungunya and dengue by means of Generalized Linear Mixed Models (GLMM), in a frequentist framework using the function *glmer.nb* implemented in the R package *lme4* (R Project for Statistical Computing, software version 4.3.2)[12].

We considered as dependent variable R_s and we assumed it to follow a Negative Binomial distribution with log link. We included in the model two qualitative independent variables: the specific disease (*D*, modeled as a binary variable: 0 for dengue and 1 for chikungunya) affecting the cases and the case classification (i.e. imported, autochthonous case with symptom onset occurring before the outbreak detection, autochthonous with symptom onset occurring after the outbreak detection; A, modeled as a dummy variable). Finally, we considered the specific outbreak (o) as a random intercept effect (a_o).

Therefore, the model equations are the following:

$$R_s \sim NegBin(\mu_s, \theta)$$
$$E(R_s) = \mu_s \; ; \; Var(R_s) = \mu_s + \mu_s{}^2/\theta$$
$$log(\mu_s) = \beta_0 + \beta_1 D_s + \beta_2 A_{before} + \beta_3 A_{after} + a_o$$
$$a_o \sim Norm(0, \sigma_2^2)$$

where $\beta_0, \beta_1,$ and β_2 are the regression parameters to be estimated along with θ.

In the analyzed dataset, we found no evidence against the hypothesis of a comparable notification delay between imported cases and autochthonous cases with symptom onset occurring after the outbreak detection nor between chikungunya and dengue cases (Table S7). On the other hand, a longer notification delay was estimated for autochthonous cases preceding the outbreak detection. Model assumptions were verified by assessment of temporal dependency and visual inspection of model residuals (Fig. S4).

Table S7. Estimated model parameters obtained by comparing notification delays of autochthonous and imported cases. The test used to compute the reported p-values is the Wald test implemented in the function *glmer.nb* from the R package *lme4*.

Regression parameters	***Estimate***	***95% Confidence Intervals***	***p-value***
Intercept (β_0)<br>Disease: dengue<br>Cases: Imported	2.252	1.952, 2.551	<0.0001
Disease: chikungunya (β_1)	0.019	-0.345, 0.382	0.91952
Cases: autochthonous preceding the outbreak detection (β_2)	0.735	0.468, 1.001	<0.0001
Cases: autochthonous following the outbreak detection (β_3)	0.049	-0.181, 0.279	0.67521
Parameter	***Estimate***		
θ	1.544		
σ_2^2	0.063		

Fig. S4. Model residuals obtained by comparing notification delays of autochthonous and imported cases.

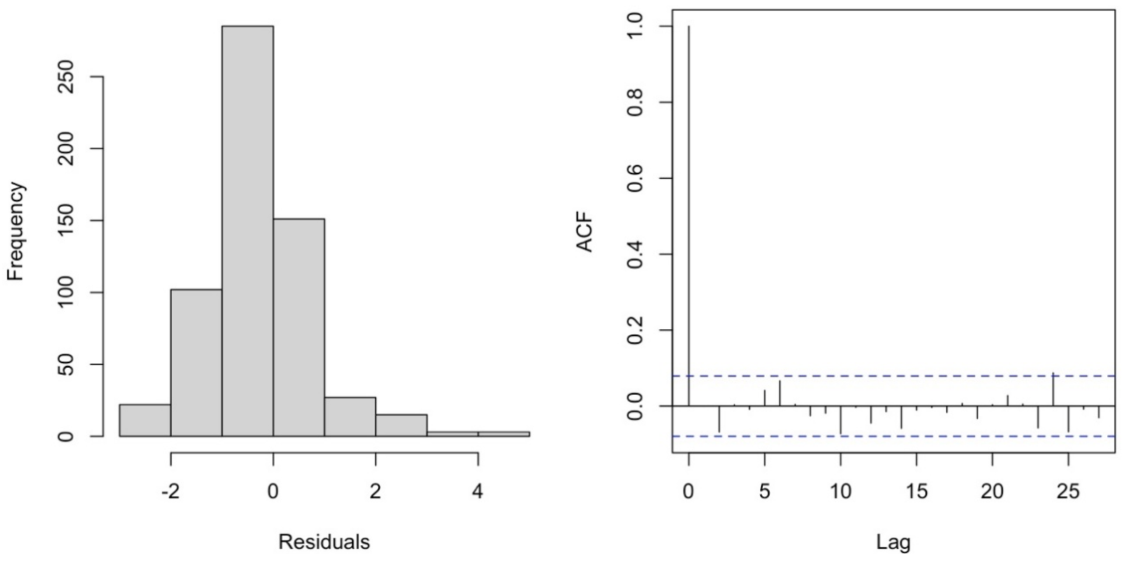

Transmissibility from surveillance of human cases

For each major outbreak occurred in Italy between 2007 and 2023, the transmissibility from surveillance of autochthonous human cases was obtained using a consolidated methodology that estimates the daily net reproduction number[13,14]. Specifically, we assumed that the daily number of new confirmed autochthonous cases (by date of symptom onset) *C(t)* can be approximated by a Poisson distribution according to the equation

$$C(t) \sim \text{Pois}\left(R_t \sum_{s=0}^{t} \varphi(s)\, C(t-s)\right)$$

where

- *C(t)*, with *t* from 1 to T, is the number of new autochthonous cases (by date of symptom onset) at day *t*;
- R_t is the net reproduction number at time *t*;
- $\varphi(s)$ is the distribution of the generation time calculated at time *s* which is assumed to follow a Gamma distribution with mean 18.3 and standard deviation 8.1 for dengue[15] and a Gamma distribution with mean 12.4 and standard deviation 4.3 for chikungunya[16].

The likelihood $\mathcal{L}$ of the observed time series of cases from day 1 to day T conditional on C(0) is thus given by

$$\mathcal{L} = \prod_{t=1}^{T} P\left(C(t);\, R_t \sum_{s=1}^{t} \varphi(s)\, C(t-s)\right)$$

where P(*k*; λ) is the probability mass function of a Poisson distribution (i.e., the probability of observing *k* events if these events occur with rate λ). The posterior distribution of R_t is estimated by using the MCMC Metropolis-Hastings sampling approach.

The risk of onward transmission and the likelihood of experiencing local outbreaks after case importation

The daily risk of onward transmission for dengue and chikungunya was estimated using the approach recently developed by Zardini et al.[17] to estimate the spatiotemporal abundance of *Aedes* mosquitoes and the consequent risk of autochthonous arboviral transmission in Europe and the Americas at a resolution of 250 m x 250 m. In the model, *Ae. albopictus* was assumed to be the competent vector species for potential arboviral transmission in Italy. The absolute number of female adults per hectare N_V at any given day d was approximated with a logistic function of the average temperature $\tilde{T}(d, w)$ observed over a certain time window w preceding that specific day:

$$N_V(d) = \alpha \frac{L}{1 + e^{-k(\tilde{T}(d,w) - T_0)}}$$

where $L, T_0,$ and k are the maximum, the midpoint, and the steepness of the logistic function while α is a scaling factor shaping how the number of estimated captures translates into a vector abundance per hectare. Model parameters were taken from Zardini et al.[17] as resulting by calibrating the model on a large set of entomological data collected across different mosquito breeding seasons and locations in Italy, Brazil, and the US.
In this study, we extended this approach by accounting for more refined data on human density (at a resolution of 100 m x 100 m) and informing the calibrated model with daily temperature records that were extracted from the E-OBS dataset at a spatial resolution of 0.1° x 0.1° (~11 km x 11 km at the equator) for the years of the analyzed outbreaks (i.e., 2007, 2017, 2020, and 2023)[18]. For the human density, we used gridded data obtained for Italy at a spatial resolution of 100 m x 100 m from the WorldPop database[19].

The model was used to assess the daily risk of onward transmission of chikungunya and dengue at a spatial resolution of 100 m x 100 m in terms of the disease-specific reproduction number R_0 and the probability that an outbreak will take place p_0. By considering a standard SEIR-SEI schema to model the host-vector transmission dynamics[20–22], these two quantities were computed as follows:

$$R_0 = R_{HV} R_{VH}$$

$$p_0 = 1 - \frac{R_{VH} + 1}{R_{VH}(R_{HV} + 1)}$$

where R_{VH} represents the average number of hosts infected by a single infective mosquito introduced in a fully susceptible human population and R_{HV} represents the average number of mosquitoes infected by the introduction of a single infective human host into a population of fully susceptible vectors. R_{VH} and R_{HV} were defined as follows:

$$R_{VH} = \beta\phi \frac{\chi_H}{\mu_V}$$

$$R_{HV} = \beta\, \phi \frac{\chi_V}{\gamma} \frac{\omega_V}{\omega_V + \mu_V} \frac{N_V}{N_H}$$

where β is the mosquito biting rate, ϕ is the proportion of mosquito blood meals taken from humans, χ_H and χ_V are the transmission probability from vector to host and from host to vector per bite respectively, μ_V represents the mortality rate of adult mosquitoes, $1/\gamma$ defines the infectious period of human hosts, $1/\omega_V$ represents the extrinsic incubation period, N_V and N_H are the number of adult female mosquitoes and the number of human individuals in a given patch. In the case of dengue, we assumed that the transmission is caused only by symptomatic infections. Therefore, to assess the associated risk of outbreaks, we considered the proportion of symptomatic infections, represented by the parameter ρ. To account for the large uncertainty surrounding this assumption, in our simulations, we explored values of ρ ranging from 0.25 to 1. All model parameters are reported in Tables S8-S9.

Table S8. Biological parameters of mosquitoes. T denotes the temperature in Celsius.

Parameter	Description	Value/Formula	Refs
μ_V	Mortality rate (day^{-1})	If $T < 15° C$<br>$\mu_V = \frac{1}{1.1 + exp(-4.04 + 0.576 * T)} + 0.12$<br>If $15 °C \leq T < 26.3 °C$<br>$\mu_V = 0.000339 * T^2 - 0.0189 * T + 0.336$<br>If $T \geq 26.3 °C$<br>$\mu_V = \frac{1}{1.065 + \exp(32.2 - 0.92 * T)} + 0.0747$	23
β	Biting rate (day^{-1})	$0.5 * (0.0043 * T + 0.0943)$	23
ϕ	human host preference	0.6	23–25

Table S9. Epidemiological parameters for chikungunya and dengue. T denotes the temperature in Celsius.

Disease	Parameter	Description	Value/Formula	Refs
chikungunya	γ^{-1}	Human infectious period (days)	$2 - 7$	26–28
	${\omega_V}^{-1}$	Mosquito extrinsic incubation period (days)	$2 - 3$	27
	χ_V	Mosquito susceptibility to infections	$70\% - 100\%$	27
	χ_H	Human susceptibility to infections	$50\% - 80\%$	27

dengue	γ^{-1}	Human infectious period (days)	$2 - 5$	29,30
	ρ	Proportion of symptomatic infections	$0.25 - 1$	31
	ω_V	Mosquito extrinsic incubation rate (day^{-1})	$1.09 * 10^{-4} * T * (T - 10.39) * (43.05 - T)^{1/2}$	32
	χ_V	Mosquito susceptibility to infections	$4.39 * 10^{-4} * T * (T - 3.62) * (36.82 - T)^{1/2}$	32
	χ_H	Human susceptibility to infections	$7.35 * 10^{-4} * T * (T - 15.84) * (36.40 - T)^{1/2}$	32

Figs. S5-S6 show model estimates of the likelihood of CHIKV and DENV onward transmission on the first day of each month in 2023. Fig. S7 shows model estimates of the DENV risk of transmission on August 1, 2023, at a 100 m x 100 m spatial resolution, compared with the exposure locations associated with an illustrative set of cases from the 2023 DENV outbreak.

Fig. S5. Model estimates of the likelihood of CHIKV onward transmission on the first day of each month in 2023. Estimates are provided at a 100 m x 100 m spatial resolution and are shown only for areas with a population density of at least ten inhabitants per hectare and where the estimated R_0 exceeds 0.1. Values are displayed as proportional to the population size to highlight potential epidemic risks and represent the average estimate of 500 model runs. Administrative boundaries of the Italian regions were retrieved from the Italian Institute of Statistics[33].

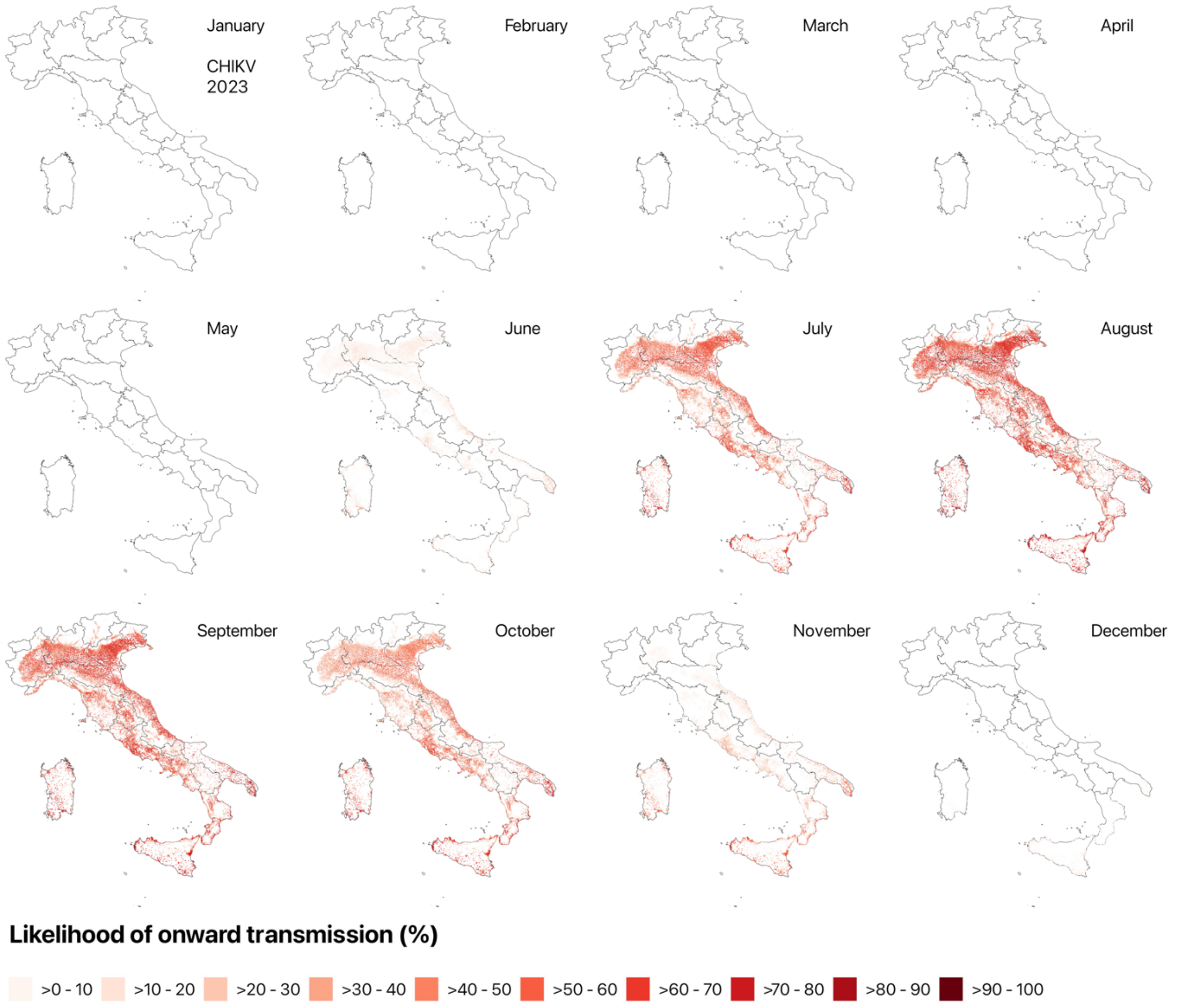

Fig. S6. Model estimates of the likelihood of DENV onward transmission on the first day of each month in 2023. Estimates are provided at a 100 m x 100 m spatial resolution and are shown only for areas with a population density of at least ten inhabitants per hectare and where the estimated R_0 exceeds 0.1. Values are displayed as proportional to the population size to highlight potential epidemic risks and represent the average estimate of 500 model runs. Administrative boundaries of the Italian regions were retrieved from the Italian Institute of Statistics[33].

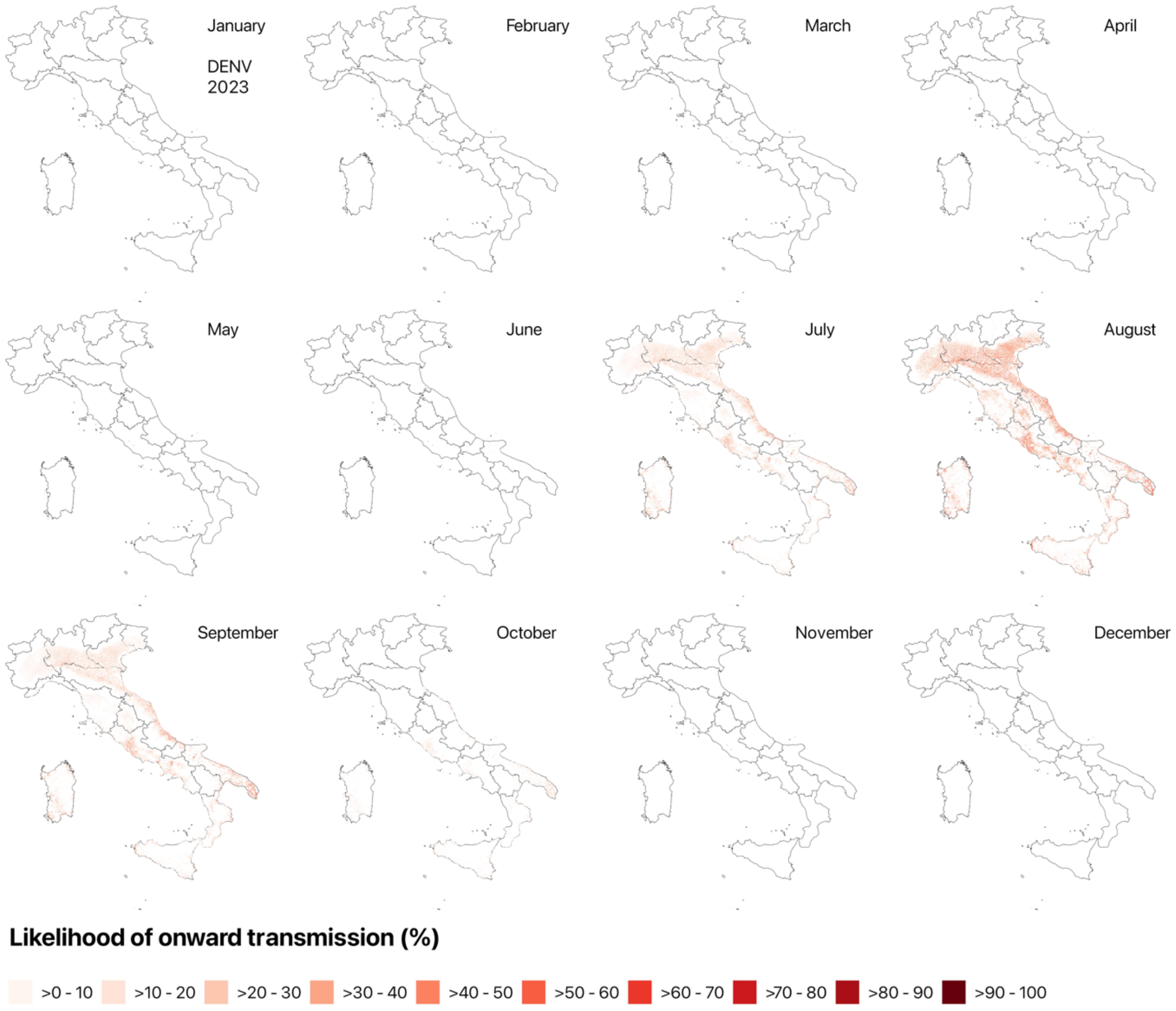

Fig. S7. Spatial heterogeneity in the transmission risks. Comparison of likely exposure locations associated with an illustrative set of cases identified during the 2023 DENV outbreak with model estimates of the DENV R_0 in Rome on August 1st, 2023. Model estimates are provided at a spatial resolution of 100 m x 100 m and are shown only for areas at risk of autochthonous transmission ($R_0 \geq 1$). Displayed values represent the average of 500 model runs. Background map layer is available under Open Database License (https://www.openstreetmap.org/copyright). © OpenStreetMap contributors.

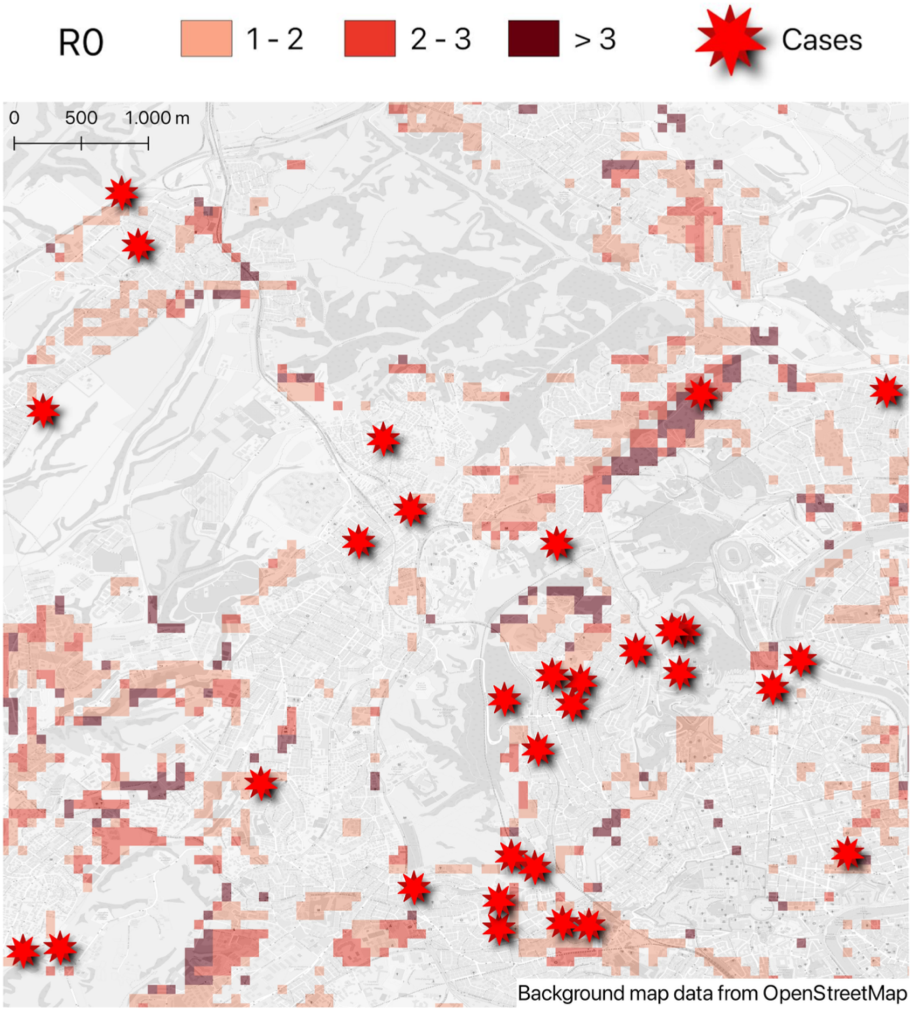

References

1. Italian Ministry of Health. National Arbovirus Plan (Piano Nazionale di prevenzione, sorveglianza e risposta alle Arbovirosi) 2020-2025. https://www.salute.gov.it/imgs/C_17_pubblicazioni_2947_allegato.pdf (2019).
2. European Centre for Disease Prevention and Control. EU case definitions. https://www.ecdc.europa.eu/en/all-topics/eu-case-definitions (2011).
3. Rezza, G. *et al.* Infection with chikungunya virus in Italy: an outbreak in a temperate region. *The Lancet* **370**, 1840–1846 (2007).
4. Edwards, C. J. *et al.* Molecular diagnosis and analysis of Chikungunya virus. *J. Clin. Virol.* **39**, 271–275 (2007).
5. Venturi, G. *et al.* Detection of a chikungunya outbreak in Central Italy, August to September 2017. *Eurosurveillance* **22**, 17–00646 (2017).
6. Merakou, C. *et al.* Diagnosis of Imported Dengue and Zika Virus Infections in Italy from November 2015 to November 2022: Laboratory Surveillance Data from a National Reference Laboratory. *Viruses* **16**, 50 (2023).
7. R. Core Team. R: a language and environment for statistical computing. R foundation for statistical computing. (2023).
8. Wood, S. N., Pya, N. & Säfken, B. Smoothing Parameter and Model Selection for General Smooth Models. *J. Am. Stat. Assoc.* **111**, 1548–1575 (2016).
9. Wood, S. N. Generalized Additive Models: An Introduction with R, Second Edition. (Chapman and Hall/CRC, New York, 2017). doi:10.1201/9781315370279.
10. Official Gazette of the Italian Republic. Decree of Italian President of Council of Ministers of 03 December 2020. [In Italian]. (2020).
11. Zuur, A. F. & Ieno, E. N. A protocol for conducting and presenting results of regression-type analyses. *Methods Ecol. Evol.* **7**, 636–645 (2016).
12. Bates, D., Mächler, M., Bolker, B. & Walker, S. Fitting Linear Mixed-Effects Models Using lme4. *J. Stat. Softw.* **67**, 1–48 (2015).
13. Manica, M. *et al.* Transmission dynamics of the ongoing chikungunya outbreak in Central Italy: from coastal areas to the metropolitan city of Rome, summer 2017. *Eurosurveillance* **22**, 17–00685 (2017).
14. Guzzetta, G., Marques-Toledo, C. A., Rosà, R., Teixeira, M. & Merler, S. Quantifying the spatial spread of dengue in a non-endemic Brazilian metropolis via transmission chain reconstruction. *Nat. Commun.* **9**, 2837 (2018).
15. Rovida, F. *et al.* The 2023 dengue outbreak in Lombardy, Italy: A one-health perspective. *Travel Med. Infect. Dis.* **64**, 102795 (2025).
16. Guzzetta, G. *et al.* Spatial modes for transmission of chikungunya virus during a large chikungunya outbreak in Italy: a modeling analysis. *BMC Med.* **18**, 226 (2020).
17. Zardini, A. *et al.* Estimating the potential risk of transmission of arboviruses in the Americas and Europe: a modelling study. *Lancet Planet. Health* **8**, e30–e40 (2024).
18. Cornes, R. C., van der Schrier, G., van den Besselaar, E. J. M. & Jones, P. D. An Ensemble Version of the E-OBS Temperature and Precipitation Data Sets. *J. Geophys. Res. Atmospheres* **123**, 9391–9409 (2018).
19. Bondarenko, M., Kerr, D., Sorichetta, A. & Tatem, A. Census/projection-disaggregated gridded population datasets for 189 countries in 2020 using Built-Settlement Growth Model (BSGM) outputs. University of Southampton https://doi.org/10.5258/SOTON/WP00684 (2020).
20. Lloyd, A. L., Zhang, J. & Root, A. M. Stochasticity and heterogeneity in host-vector models. *J. R. Soc. Interface* **4**, 851–863 (2007).

21. Guzzetta, G. *et al.* Potential Risk of Dengue and Chikungunya Outbreaks in Northern Italy Based on a Population Model of Aedes albopictus (Diptera: Culicidae). *PLoS Negl. Trop. Dis.* **10**, e0004762 (2016).
22. Guzzetta, G. *et al.* Assessing the potential risk of Zika virus epidemics in temperate areas with established Aedes albopictus populations. *Eurosurveillance* **21**, 30199 (2016).
23. Caminade, C. *et al.* Global risk model for vector-borne transmission of Zika virus reveals the role of El Niño 2015. *Proc. Natl. Acad. Sci. U. S. A.* **114**, 119–124 (2017).
24. Faraji, A. *et al.* Comparative host feeding patterns of the Asian tiger mosquito, Aedes albopictus, in urban and suburban Northeastern USA and implications for disease transmission. *PLoS Negl. Trop. Dis.* **8**, e3037 (2014).
25. Sivan, A., Shriram, A. N., Sunish, I. P. & Vidhya, P. T. Host-feeding pattern of Aedes aegypti and Aedes albopictus (Diptera: Culicidae) in heterogeneous landscapes of South Andaman, Andaman and Nicobar Islands, India. *Parasitol. Res.* **114**, 3539–3546 (2015).
26. Massad, E. *et al.* The risk of chikungunya fever in a dengue-endemic area. *J. Travel Med.* **15**, 147–155 (2008).
27. Poletti, P. *et al.* Transmission potential of chikungunya virus and control measures: the case of Italy. *PloS One* **6**, e18860 (2011).
28. Manore, C. A., Hickmann, K. S., Xu, S., Wearing, H. J. & Hyman, J. M. Comparing dengue and chikungunya emergence and endemic transmission in A. aegypti and A. albopictus. *J. Theor. Biol.* **356**, 174–191 (2014).
29. Marini, G. *et al.* Effectiveness of Ultra-Low Volume insecticide spraying to prevent dengue in a non-endemic metropolitan area of Brazil. *PLoS Comput. Biol.* **15**, e1006831 (2019).
30. Brady, O. J. *et al.* Global temperature constraints on Aedes aegypti and Ae. albopictus persistence and competence for dengue virus transmission. *Parasit. Vectors* **7**, 338 (2014).
31. Ferguson, N. M. *et al.* Benefits and risks of the Sanofi-Pasteur dengue vaccine: Modeling optimal deployment. *Science* **353**, 1033–1036 (2016).
32. Mordecai, E. A. *et al.* Detecting the impact of temperature on transmission of Zika, dengue, and chikungunya using mechanistic models. *PLoS Negl. Trop. Dis.* **11**, e0005568 (2017).
33. Italian National Institute of Statistics (ISTAT). Administrative boundaries for statistical use (Confini delle unità amministrative a fini statistici). https://www.istat.it/notizia/confini-delle-unita-amministrative-a-fini-statistici-al-1-gennaio-2018-2/ (2025).
