# Supplementary material for: Risk assessment and perspectives of local transmission of chikungunya and dengue in Italy, a European forerunner
Source: Nat Commun. 2025 Jul 7;16:6237. doi: 10.1038/s41467-025-61109-1 (PMC12234714; doi:10.1038/s41467-025-61109-1)
Supplement: Supplementary file 2 — Reporting Summary [file 41467_2025_61109_MOESM2_ESM.pdf]

Corresponding author(s): Piero Poletti
Last updated by author(s): May 21, 2025

Reporting Summary

Nature Portfolio wishes to improve the reproducibility of the work that we publish. This form provides structure for consistency and transparency in reporting. For further information on Nature Portfolio policies, see our Editorial Policies and the Editorial Policy Checklist.

Statistics

For all statistical analyses, confirm that the following items are present in the figure legend, table legend, main text, or Methods section.

n/a	Confirmed	
☐	☒	The exact sample size (*n*) for each experimental group/condition, given as a discrete number and unit of measurement
☒	☐	A statement on whether measurements were taken from distinct samples or whether the same sample was measured repeatedly
☐	☒	The statistical test(s) used AND whether they are one- or two-sided<br>*Only common tests should be described solely by name; describe more complex techniques in the Methods section.*
☐	☒	A description of all covariates tested
☐	☒	A description of any assumptions or corrections, such as tests of normality and adjustment for multiple comparisons
☐	☒	A full description of the statistical parameters including central tendency (e.g. means) or other basic estimates (e.g. regression coefficient) AND variation (e.g. standard deviation) or associated estimates of uncertainty (e.g. confidence intervals)
☐	☒	For null hypothesis testing, the test statistic (e.g. *F*, *t*, *r*) with confidence intervals, effect sizes, degrees of freedom and *P* value noted<br>*Give P values as exact values whenever suitable.*
☐	☒	For Bayesian analysis, information on the choice of priors and Markov chain Monte Carlo settings
☒	☐	For hierarchical and complex designs, identification of the appropriate level for tests and full reporting of outcomes
☒	☐	Estimates of effect sizes (e.g. Cohen's *d*, Pearson's *r*), indicating how they were calculated

Our web collection on statistics for biologists contains articles on many of the points above.

Software and code

Policy information about availability of computer code

Data collection	Case data were collected by the Italian arbovirus surveillance system. Temperature data were downloaded from the E-OBS dataset, a collection of high-resolution gridded climate data covering Europe and part of the Copernicus Climate Change Service (https://surfobs.climate.copernicus.eu/dataaccess/access_eobs.php). Population data were downloaded for Italy from the Worldpop database (https://hub.worldpop.org/geodata/summary?id=49809).
Data analysis	Case data were analyzed using R Project for Statistical Computing (software version 4.3.2). The code will be made available in Zenodo upon publication at 10.5281/zenodo.15100955. The estimation of reproduction numbers and the risk of onward transmission was performed using codes developed ad-hoc by our team in the programming language C, available at https://zenodo.org/records/10203374. Maps were created using QGIS software version 3.30.2.

For manuscripts utilizing custom algorithms or software that are central to the research but not yet described in published literature, software must be made available to editors and reviewers. We strongly encourage code deposition in a community repository (e.g. GitHub). See the Nature Portfolio guidelines for submitting code & software for further information.

Data

Policy information about availability of data

All manuscripts must include a data availability statement. This statement should provide the following information, where applicable:

- Accession codes, unique identifiers, or web links for publicly available datasets
- A description of any restrictions on data availability
- For clinical datasets or third party data, please ensure that the statement adheres to our policy

The data contain confidential information, and public data deposition is not permitted. Due to the sensitive nature of the data, raw data can only be made available by the Istituto Superiore di Sanità (Italian National Institute of Health) through a data-sharing agreement directly with the user (contact mail: sorveglianza.arbovirosi@iss.it).Aggregated data are available at https://www.epicentro.iss.it/arbovirosi/dashboard and https://www.epicentro.iss.it/arbovirosi/bollettini.

Research involving human participants, their data, or biological material

Policy information about studies with human participants or human data. See also policy information about sex, gender (identity/presentation), and sexual orientation and race, ethnicity and racism.

Reporting on sex and gender	Analyzed data consists of epidemiological surveillance records. Information on sex was collected by the Italian surveillance system for each case (935 males and 1,122 females). The study analyzed the importation of dengue and chikungunya cases in Italy and the risk of onward transmission regardless sex and gender distinction, therefore no sex- and gender-based analyses were conducted.
Reporting on race, ethnicity, or other socially relevant groupings	Analyzed data consists of epidemiological surveillance records. No information on race, ethnicity, or other social relevant grouping was included.
Population characteristics	Analyzed data consists of epidemiological surveillance records. Analyzed variables for each reported case included: pathogen laboratory confirmation, date of symptom onset, date of notification, age, sex, the list of clinical symptoms experienced by the case, classification of cases as travel-related or locally acquired, and geolocation of likely exposure for autochthonous cases and the country of likely exposure as identified during epidemiological interviews.
Recruitment	Data on cases were collected by the Italian surveillance system, thus it did not involve recruitment.
Ethics oversight	This study was conducted using epidemiological data from the Italian national integrated arbovirus surveillance routinely collected and analyzed within the mandate of the Italian National Institute of Health; therefore, no ethical approval was necessary.

Note that full information on the approval of the study protocol must also be provided in the manuscript.

Field-specific reporting

Please select the one below that is the best fit for your research. If you are not sure, read the appropriate sections before making your selection.

☒ Life sciences ☐ Behavioural & social sciences ☐ Ecological, evolutionary & environmental sciences

For a reference copy of the document with all sections, see nature.com/documents/nr-reporting-summary-flat.pdf

Life sciences study design

All studies must disclose on these points even when the disclosure is negative.

Sample size	The analysis was conducted on 1,577 laboratory confirmed travel-related cases and 481 autochthonous cases reported between 2006 and 2023 to the Italian database for human surveillance system for arboviral infections.
Data exclusions	64 travel-related cases and 10 autochthonous cases were excluded due to data incompleteness or inconsistencies between the dates of symptom onset and notification.
Replication	The codes used in this manuscript have been run by different users on multiple computers, providing reproducible results.
Randomization	Randomization is not relevant for this study.
Blinding	Blinding is not relevant for this study.

Reporting for specific materials, systems and methods

We require information from authors about some types of materials, experimental systems and methods used in many studies. Here, indicate whether each material, system or method listed is relevant to your study. If you are not sure if a list item applies to your research, read the appropriate section before selecting a response.

Materials & experimental systems

n/a	Involved in the study
☒	☐ Antibodies
☒	☐ Eukaryotic cell lines
☒	☐ Palaeontology and archaeology
☒	☐ Animals and other organisms
☒	☐ Clinical data
☒	☐ Dual use research of concern
☒	☐ Plants

Methods

n/a	Involved in the study
☒	☐ ChIP-seq
☒	☐ Flow cytometry
☒	☐ MRI-based neuroimaging

Plants

Seed stocks	*Report on the source of all seed stocks or other plant material used. If applicable, state the seed stock centre and catalogue number. If plant specimens were collected from the field, describe the collection location, date and sampling procedures.*
Novel plant genotypes	*Describe the methods by which all novel plant genotypes were produced. This includes those generated by transgenic approaches, gene editing, chemical/radiation-based mutagenesis and hybridization. For transgenic lines, describe the transformation method, the number of independent lines analyzed and the generation upon which experiments were performed. For gene-edited lines, describe the editor used, the endogenous sequence targeted for editing, the targeting guide RNA sequence (if applicable) and how the editor was applied.*
Authentication	*Describe any authentication procedures for each seed stock used or novel genotype generated. Describe any experiments used to assess the effect of a mutation and, where applicable, how potential secondary effects (e.g. second site T-DNA insertions, mosiacism, off-target gene editing) were examined.*
